# Hybrid Cyber Petri net Modelling, Simulation and Analysis of Master-Slave Charging for Wireless Rechargeable Sensor Networks

**DOI:** 10.3390/s21020551

**Published:** 2021-01-14

**Authors:** Huaiyu Qin, Buhui Zhao, Leijun Xu, Xue Bai

**Affiliations:** 1 School of Electrical and Information Engineering, Jiangsu University, Zhenjiang 212000, China; qhy@just.edu.cn (H.Q.); zhaobuhui@ujs.edu.cn (B.Z.); xlking@ujs.edu.cn (L.X.); 2 School of Electrical and Information Engineering, Jiangsu University of Science and Technology, Zhenjiang 212000, China

**Keywords:** wireless charging, hybrid cyber petri net, master-slave charging, wireless rechargeable sensor network

## Abstract

Wireless charging provides continuous energy for wireless sensor networks. However, it is difficult to replenish enough energy for all sensor nodes with fixed charging alone, and even more unrealistic to charge a large number of nodes within a short time via mobile charging. In order to overcome the above weaknesses, this paper firstly puts forward a Master-Slave Charging mode for the WRSN (Wireless Rechargeable Sensor Network), where fixed charging is the master mode and mobile charging is the slave mode, respectively. However, Master-Slave Charging is a typical hybrid system involving discrete event decision and continuous energy transfer. Therefore, the Hybrid Cyber Petri net system is proposed to build a visual specification with mathematical expression of Master-Slave Charging. Moreover, wireless charging in the WRSN is modeled and evaluated from the perspective of a hybrid system for the first time. Furthermore, a greedy-genetic algorithm is proposed to obtain the deployment of fixed chargers and the path planning of a mobile charger, by maximizing the actual electric quantity of the master charging problem and minimizing the mobile charger’s travelling path of the slave charging problem. Finally, the simulation results confirm and verify the Hybrid Cyber Petri net model for Master-Slave Charging. It is worth noting that the proposed model in this paper is highly adaptable to various charging modes in the WRSN.

## 1. Introduction

With the development of wireless charging, the Wireless Rechargeable Sensor Network (WRSN) has become an important research field, which is widely used in environmental monitoring and protection, industrial manufacturing, intelligent transportation and so on. In traditional wireless sensor networks, limited energy of nodes’ batteries seriously restricts the life of the network and bring a high maintenance cost. This bottleneck is broken through by wireless charging technology, which makes the life of the network close to infinite theoretically. There are three main ways to realize it, namely electromagnetic inductive coupling, magnetic resonance coupling and RF (radio frequency) energy harvesting. Electromagnetic induction works like a transformer in which the current is generated by coupling between the primary and secondary coils, thus transferring energy from the sender to the receiver. The magnetic resonance exchange of energy is achieved by tuning two coils with the capacitance to resonate at the same frequency. Both of the above two techniques are near-field wireless transmission, and the transmission efficiency depends on the distance between two coils. Besides, both inductive coupling and resonance coupling require calibration and alignment of coils. In contrast, RF energy transfer has no such limitation, which can be regarded as a far-field technique. In RF energy harvesting, radio signals, with frequency range from 300 GHz to as low as 3 kHz, are used as a medium to carry energy in a form of electromagnetic radiation. Therefore, RF energy transfer is suitable for the WRSN [1,2,3].

There are two main types of power carrier: fixed charger and mobile charger. The former is easy to maintain and can effectively supplement the energy of sensor nodes within the radiation radius, yet it is difficult to replenish enough power outside the radiation. It is not worth adding fixed chargers for few nodes from the economic perspective. The latter generally refers to an intelligent car or an UAV (unmanned aerial vehicle) with a charger, which is more flexible. Nevertheless, the disadvantage is that when there are a large number of sensor nodes to be charged, the charging delay is difficult to be eliminated, and the energy cannot be replenished in time, resulting in the failure of network eventually. Most of the previous work was based on the separate form of fixed or mobile charger. However, the inherent characteristics of both charging modes make it impossible to fundamentally solve the problems mentioned above [4,5,6,7,8,9].

In order to release potential abilities of both types, a joint charging scheme is innovatively proposed. Fixed charging is to meet the energy of most nodes as the master pattern, while mobile charging is to take turns charging the residual nodes as the slave pattern. Although the benefit of this method is obvious, it is extremely challenging. Firstly, how to allocate the nodes for fixed or mobile charging? Secondly, what is the number of fixed chargers? On the one hand, more chargers can improve the charging effect, which will lead to higher deployment costs and energy waste; on the other hand, too few fixed chargers will increase the number of nodes that are not fully charged, thereby aggravating the burden of the mobile charger. Moreover, the sensor nodes can harvest more energy with optimal location of chargers. Thirdly, due to limited energy of the mobile charger, the other challenge is to short its moving distance as much as possible. For this purpose, Master-Slave Charging (MSC) is constructed. Firstly, the growth rate of actual electric quantity is adopted to determine the deployment scale of fixed chargers, and the maximization of actual energy received by nodes is taken as the objective to obtain the optimal location. Secondly, the slave charging will be transformed into a traveling salesman problem in order to minimize the travel path of the mobile charger. Therefore, a greedy genetic algorithm is proposed to solve MSC.

Master-Slave Charging is a typical hybrid system involving discrete event decision and continuous energy transfer. Therefore, the Hybrid Cyber Petri net System (HCPNS) is presently used for the purpose of describing wireless charging’s energy flow, data flow and control flow mathematically and visually. In addition, this is a formal specification with continuous time layer, discrete time layer and discrete event layer. Moreover, the ultimate model is different from the one shown in our previous work [10]. The charging behavior and charging process are both formalized by the new model. To the best of our knowledge, no previous work has been done to model and evaluate wireless charging for WRSN from the perspective of a hybrid system.

The main contribution focuses on constructing, modelling and solving Master-Slave Charging:

(1) Considering the disadvantages of single fixed or mobile charging, we propose a new charging paradigm. The number and location of fixed chargers are constrained by the growth rate of actual electric quantity, and the optimization goal is to maximize the total actual electric quantity of sensor nodes with fixed charging. Then, a mobile charger takes turns charging the remaining nodes with the optimization goal of minimizing the travelling distance.
(2) Wireless charging for the WRSN is a hybrid system including discrete event decision and continuous energy transfer, in which there are energy flow, data flow and control flow relationships. In addition, a formal specification with graph-mathematical characterization ability is beneficial to the analysis and application of such system. As a result, the Hybrid Cyber Petri net System is proposed based on the classical Petri net to model the Master-Slave Charging.
(3) Because Master-Slave Charging is NP-complete, it is difficult to solve using the traditional optimization method. A greedy-genetic algorithm is proposed by adding greedy crossover and mutation operators in a genetic algorithm, which enhances the local search capability and can be used for reference to solve the chargers’ scheduling problem.

The rest of this paper is organized as follows. Section 2 reviews the related works. Section 3 describes the wireless charging model and the Hybrid Cyber Petri net System. Section 4 formulates Master-Slave Charging with the HCPNS, and a greedy-genetic algorithm is applied for solving it. Section 5 shows and analyzes the numerical simulation results. Section 6 concludes this paper.

## 2. Related Work 

This section presents a brief of overview of wireless charging for the WRSN and the application of Petri net. 

### 2.1. Petri Net

We proposed GSCCPNs (Generalized Synchronizing Colored Cyber Petri nets) to model the fixed charging in 3D scenario, optimizing the number, transmitting power and deployment location of chargers [10]. J.S. Lee put forward a command filtering framework accepting or rejecting the human-issued commands to prevent the undesirable executions using Petri net modeling [11]. In order to balance the energy load of sensor nodes and provide global reliability in wireless sensor networks, Yu et al. proposed a reliable energy-efficient multi-level routing algorithm based on fuzzy Petri nets, which was employed to select cluster nodes [12]. M.C. Ruiz et al. used applied Petri nets to model the routing algorithm based on network roles, and evaluated the algorithm with state space analysis and performance evaluation [13]. Later, they used time-colored Petri nets to model the power consumption of nodes in IoT, and to save energy in Bluetooth Low-Energy (BLE) devices with optimization of the sleep state [14]. Mostafa, A. et al. presented EHFP (Energy Harvesting aware clustering with Fuzzy Petri net algorithm) for selecting cluster heads based on Fuzzy Petri net, which considered the harvested energy, number of neighbors and the centrality during clustering of the wireless sensor network (WSN) [15]. To ensure there is no deadlock, no dataflow and no mutual exclusion in discrete event system, J.C.M. Moreno et al. modeled and analyzed the discrete event system using colored Petri nets, and established a set of design methods for the shortest timing sequence of task execution [16]. R. Abrishambaf et al. utilized the Petri net as an analytical tool for event-based systems for the performance of the structural and behavioral models [17]. Aiming at the nonuniformity of nodes’ power consumption in the wireless sensor network, Jiang, F.C. et al. used Petri nets and comprehensive mathematical tools, respectively, to conduct qualitative and quantitative analysis on all relevant parties of the configuration system [18]. Berrachedi, A used a deterministic stochastic Petri net for system modeling to reduce the energy consumption of nodes and the packet loss ratio in Wireless Sensor Networks (WSNs). Moreover, in order to illustrate the applicability of the model, a special routing technology for WSNs is proposed [19]. Kostin, A.E. et al. proposed a routing scheme for WSNs with mobile sensors and mobiles multiple sinks, which was investigated with a detailed simulation model, and implemented in terms of a class of extended Petri nets [20]. Le K. et al. proposed a tool, known as CODE-WSN (Congestion Detection on WSN) to detect possible congestion occurrence, which was modeled by the formal modelling language Petri net, to allow one to inspect and verify congestion potential [21]. Riouali, Y. et al. brought forward a road traffic management system based on WSNs, introduced the functional and deployment architecture of the system and focused on the analysis component that used a new extension of batched Petri nets for modeling road traffic flow [22]. Mahjoub, Y.I. et al. proposed a Timed Colored Petri net (TCPN) and (max, +) algebra-based approach to model and evaluate the performances of a bus network characterized by complex phenomena such as conflicts, synchronization and concurrency [23].

Although Petri nets have been applied in WSNs, few models were involved in sensor nodes’ energy, control flow and dataflow. Moreover, there is no unified model of visualization and mathematics. The Petri net model proposed in this paper is the first time wireless charging in the WRSN is modelled from the perspective of a hybrid system, which is adaptive to other charging systems. 

### 2.2. Wireless Charging

Many scholars have done fruitful work on wireless charging for the WRSN. He et al. divided the charger deployment problem into static and dynamic, which involved point and path provisioning, respectively. Both of them were deployed in an equilateral triangle way, and the number of chargers could be reduced as far as possible by expanding the triangle area. The point provisioning is proved to be able to achieve sub-optimal performance, and the path provisioning is actually close to the optimal performance [24]. Jiang et al. modeled the charging space as a cone and took candidate cones as possible deployment locations. They proposed greedy cone covering algorithm and adaptive cone covering algorithm to obtain the minimum number of candidate cones [25]. Then, they proposed the Evolutionary Beamforming Optimization Reseeding (EBO-R) algorithm to nearly optimize the power ratio of the uniform circular array beamforming Peak Side Lobe (PSL) and the Main Lobe (ML) aimed at the given target direction [26]. To solve the Wireless Charging Nodes (WCNs) deployment optimization problem, Sun et al. presented an Improved Firefly Algorithm (IFA), which adopts a novel adaptive attractiveness factor, and introduced a dynamic location update mechanism to enhance the performance of the normal firefly algorithm [27]. Meanwhile they proposed an optimization framework that simultaneously maximizes the coverage and the charging efficiency, which outperformed other comparative algorithms in both accuracy and convergence rate [28]. Dai et al. put forward the Safe Charging Problem (SCP) with adjustable power, which maximize the charging utility within the given threshold [29,30]. Later, they studied the radiation constrained scheduling in [31]. Wan et al. proposed a new algorithm based on the greedy algorithm and the location relationship of the sensor nodes, which exploited the local search ability and avoids falling into an exponential increase of the number of base stations [32].

However, most of the work overlooks the capacity of nodes’ batteries. Sensor nodes cannot receive the energy from the charger indefinitely. Hence, the redundant energy is useless. In this paper, we apply the actual electric quantity as the basis for the deployment of fixed chargers.

In terms of mobile charging, Wang et al. in [33] used charging vehicles and dedicated data collection vehicles to perform charging and data collection simultaneously. On the basis of [33], they proposed to combine solar energy collection with mobile charging to reduce the cost of mobile chargers [34]. Since previous mobile charging protocols focused on either the charger travel distance or the charging delay of sensor nodes, Fu et al. proposed a novel Energy Synchronized (ESync) mobile charging protocol, which simultaneously reduces the charger travel distance and the charging delay [35]. To address the operation scheduling problem when improving the performance of charging and communication, Shu et al. proposed a joint energy replenishment and scheduling mechanism so as to maximize the network lifetime while making strict sensing guarantees in the WRSN [36]. In [37], it was assumed that chargers’ velocity can be adjusted, they formulated the optimal charger velocity control framework on arbitrarily-shaped irregular trajectories in a 2D space and developed a heuristic solution to maximize the charged energy in sensor nodes. Extending wireless power transfer to the underwater environment, Lin et al. used charging robot mules to replenish the energy. They developed a Shortest-Path Charging Scheme (SCS) to minimize the traveling cost for the charging mules and an Emergency Charging Scheme (ECS) to concentrate service to emergency nodes. After that, SCS and ECS were combined to collaboratively solve the charging problem [38]. Different from traditional charging scheduling policies where sensor nodes passively wait for the arrival of mobile vehicles, a novel dynamic clustering based on the Mobile-To-Cluster (M2C) scheme was proposed to optimize the service process for both sensor nodes and the vehicle in an active way. Requiring only local residual energy information, M2C refreshes the cluster-based network topology to ensure no sensor nodes will run out of energy before getting charged [39]. Sheikhi et al. put forward an approach named Limited Knowledge Charging (LKC) to prolong the network lifetime and reduce the movements of mobile chargers [40]. Ai et al. proposed a novel distributed mobile charging algorithm, to optimize the charging time and chargers’ quantity, and an improved algorithm, named adaptive dynamic energy transfer, to further promote the network capacity in 5G networks [41]. Mo et al. addressed the multiple Mobile Chargers (MCs) coordination problem under multiple requirements, which jointly optimized the scheduling, moving time and charging time of MCs [42]. Lyu et al., in [43], divided the network into multiple cells, and proposed a periodic multi-node charging and data collection scheme to maintain a perpetual operation. According to the importance of the sensor node, which was associated with the distance to the base station, Jiao et al. in [44], divided sensor nodes into two types: Sensor nodes close to the base station and sensor nodes far away from the base station. In addition, different charging methods were adopted by the mobile wireless charging vehicle. Different from their method, this paper considers the location, residual energy and power consumption of sensor nodes together to determine the fixed charging nodes set, and further obtains the mobile charging nodes set. 

Based on the above research, the deployment is vulnerable to restrictions such as terrain, personal safety and other factors, hence it is difficult to achieve the ideal charging effect only via fixed chargers. Although mobile charging is more flexible, its optimization scheduling mechanism is complex and difficult to maintain a large number of mobile chargers. For this reason, this paper proposes a Master-Slave Charging, whereby fixed and mobile charging are master mode and slave mode, respectively. 

## 3. System Model

In this section, the model of energy consumption and wireless charging is first introduced, and then the Hybrid Cyber Petri net System (HCPNS) is proposed on the basis of ordinary Petri net, in order to model the wireless charging for the WRSN simultaneously from mathematical and visual perspectives.

### 3.1. Syetem Model of Wireless Charging 

**Figure 1:**
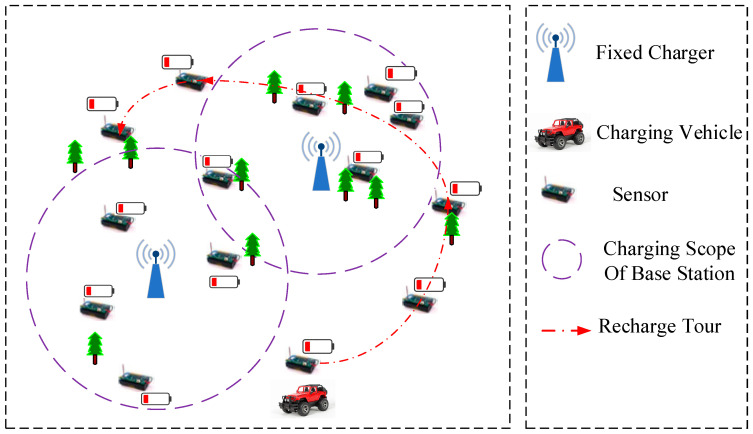
Illustration of the network architecture and components.

The system structure is shown in Figure 1, which consists of three parts: fixed chargers, a mobile charger and sensor nodes. Considering a WRSN with *n* sensor nodes and m chargers, the nodes within the radiation range are charged by master mode (fixed charging), while others are charged by slave mode (mobile charging). A set of nodes by fixed charging and mobile charging is $\left\{s_{1},\ldots ,s_{j}\right\}$ and $\left\{s_{j+1},\ldots ,s_{n}\right\}$, respectively. A set of fixed chargers is $\left\{c_{1},\ldots ,c_{m-1}\right\}$, and $c_{m}$ is a mobile charger. The main notations are summarized in Table 1.

For each node $s_{p}\left(p=1,2,\cdots ,n\right)$, its energy is mainly consumed for the data transmitting and receiving. The energy consumption model is as follows [45]:

(1) $$r_{p}=\rho \sum_{k=1,k\neq p}^{n}f_{kp}+\sum_{l=1,l\neq p}^{n}C_{pl}f_{pl}+C_{pb}f_{pb}$$

where $f_{kp}$ ($f_{pl}$) and $f_{pb}$ are the data flow from node $s_{k}$ to node $s_{p}$ (from node $s_{p}$ to node $s_{l}$) and from node $s_{p}$ to base station (BS), respectively. ρ and $C_{pl}$ ($C_{pb}$) are the rate of energy consumption for receiving a unit of data and transmitting a unit of data from node $s_{p}$ to node $s_{l}$ (BS), respectively. It is assumed that the system runs with a given routing protocol, thus the energy consumption rate $r_{p}$ of node $s_{p}$ is invariant with time.

On the other hand, the following wireless charging model is employed [24]:

(2) $$p_{r}\left(s_{p},c_{i}\right)=\{\begin{array}{ll} \frac{\alpha }{{\left(d_{ip}+\beta \right)}^{2}}, & d\leq r\\ 0, & d>r \end{array}$$

where $\alpha =\frac{\left(G_{s}G_{r}\eta \lambda^{2}\cdot p_{f}\right)}{\left(16L_{p}\pi^{2}\right)}$, $p_{f}$ is the source power of a charger, $p_{r}$ is the received power of a node, $d_{ip}$ is the distance between the charger $c_{i}$ and node $s_{p}$, $G_{s}$ and $G_{r}$ is the source antenna gain and receive antenna gain, $L_{p}$ is polarization loss, λ is wave length, r is the effective radiation radius, and η can be referred to as rectifier efficiency.β is a parameter to adjust the Friis’ free space equation. $p_{r}$ is the received power of a node. 

It is believed that a fixed charger can charge multiple sensor nodes at the same time, and the power received by one sensor node can also be added. The total power of $s_{p}$ received from $c_{i}$ is:

(3) $$p_{r}\left(s_{p}\right)=\sum_{i=1}^{m-1}p_{r}\left(s_{p},c_{i}\right)$$

Every node receives different amounts of energy at a given time due to the distance from the fixed charger. For a node $s_{p}$, if the received energy $E_{r}\left(s_{p}\right)$ is larger than the sum of its consumption energy $E_{c}\left(s_{p}\right)=r_{p}\cdot T$ and its energy needed to be full $E_{need}\left(s_{p}\right)$, this is overcharging. On the one hand, as long as the node receives no less energy than it consumes, it is acceptable even if not full. On the other hand, since the battery capacity of a node is finite, overcharging is unprofitable and meaningless. Therefore, actual electric quantity is defined as follows.

**Definition** **1.** **Actual Electric Quantity (AEQ).***To replenish full energy of*$s_{p}$*, the least energy received is the sum of consumption energy *$E_{c}\left(s_{p}\right)$*before deadline T and energy to be full*$E_{need}\left(s_{p}\right)$*. So AEQ is defined as the smaller value between the sum of*$E_{c}\left(s_{p}\right)$, $E_{need}\left(s_{p}\right)$*and received energy*$E_{r}\left(s_{p}\right)$*. It reflects the actual valuable energy, and the CQ of*$s_{j}$*is*

(4) $$Q\left(s_{p}\right)=min\left[E_{r}\left(s_{p}\right),E_{c}\left(s_{p}\right)+E_{need}\left(s_{p}\right)\right]$$

**Definition** **2.** **Growth Rate of AEQ.** *The definition refers to the growth rate obtained by adding a charger, that is*

(5) $$\delta =\frac{Q\left(s,\left\{c_{1}\cdots c_{i-1}\right\}\cup \left\{c_{i}\right\}\right)-Q\left(s,\left\{c_{1}\cdots c_{i-1}\right\}\right)}{Q\left(s,\left\{c_{1}\cdots c_{i-1}\right\}\right)}$$

The larger the growth rate is, the better the charging revenue, and vice versa.

There is no overcharging for mobile charging, because the mobile charger is to go to the next node or to the BS as long as it achieves the service for a node. Moreover, it is assumed that the mobile charger services the sensor nodes one by one, and the distance between them is zero.

### 3.2. Petri Net Model

In its basic form, a Petri net is a directed bipartite graph composed of one set of places (drawn as circles) and transitions (drawn as bars). Places may contain tokens which are drawn as dots. Places and transitions are connected by directed arcs. Arcs may be labeled with integer numbers denoting their weights. A transition is said to be enabled, if all of its input places contain at least as many tokens as the weight of the corresponding input arc. A transition fires by removing as many as tokens from each input places as the weight of the corresponding input arc, and by adding as many as tokens to each output place as the weight of the corresponding output arc [46]. Formally, the definition of Petri Net is given below.

**Definition 3.**  *A triple* N=(S,T;F) *is said to be **Directed net** (referred to as net), if it satisfies the following conditions:*

*(1)*  S∩T=Φ∧S∪T≠Φ;
*(2)*  F⊆S×T∪T×S;
*(3)*  dom(F)∪cod(F)=S∪T *.*

*where:* S *and* T *is said to be the place set and transition set of* N *respectively,* F *is the flow relationship;* dom(F)={x|∃y:(x,y)∈F} *is the definitional domain;* cod(F)={y|∃x:(x,y)∈F} *is the value domain of* N *;* x•={y|y∈X∧(y,x)∈F} *is the input (pre-set) of* x *;* $x^{•}=\left\{y|y\in X\wedge \left(x,y\right)\in F\right\}$ *is the output (post-set) of* x *.*

**Definition 4.**  *A sextuple* $\sum =\left(S,T;F,K,W,M_{0}\right)$ *is said to be a **Petri net System**, if it satisfied the following conditions:*

*(1)*  N=(S,T;F)*is a directed net, which is named the basic net of*∑ ;
*(2)*  K:S→{1,2,3,⋯}∪{∞}*is the capacity function of*N;
*(3)*  W:S→{1,2,3,⋯} *is the weight function of* N *;*
*(4)*  ∀s∈S,M(s)≤K(s) *,* M:S→{1,2,3,⋯}M:S→{1,2,3,⋯} *is the marking, and* $M_{0}$ *is the initial marking.*

**Definition 5.**  *A 5-tuple* $\sum =\left(S,T;F,W,M_{0}\right)$ *is said to be a **Cyber net System**, if it satisfied the following conditions:*

*(1)*  N=(S,T;F) *is a directed net, which is named the basic net of* ∑ *;*
*(2)*  W:S×T∪T×S→{0,1,2,⋯}∪S *is the weight function of* ∑ *,* W(x,y)≠0 *, if and only if* (x,y)∈F *;*
*(3)*  $M_{0}:S\to \left\{0,1,2,\cdots \right\}$ *is the initial marking.*

It is can be seen that, from Definition 5, place set is added to the value domain of weight function, so the weight of directed arc is changed from a constant to a variable. Compared with the Petri net System, the Cyber net System can represent the system parameters more clearly, so as to reflect the more complex coupling relationship.

**Definition 6.**  $A=\left(a_{ij}\right)$ *is said to be the **incidence matrix** of a Petri net System, where* $a_{ij}=W\left(t_{j},s_{i}\right)-W\left(s_{i},t_{j}\right)$ *.*

**Definition 7.**  *A sextuple* $\sum =\left(S,T;F,K,W,M_{0}\right)$ *is said to be a **Generalized Cyber P/N System** if it satisfied the following conditions:*

*(1)*  N=(S,T;F) *is a directed net, which is named the basic net of* ∑ *;*
*(2)*  $K=\left\{k_{L},k_{H}\right\}$ *is the capacity function of* N *, and* $k_{L}:S\to ℜ;k_{H}:S\to ℜ$ *,where* ℜ *is a set of real numbers;* $k_{L}$ *and* $k_{H}$ *is lower and upper of* K *;*
*(3)*  W:F→ℜ∪Exp(S) *is the weight function, where* Exp(S) *is the set of functional expressions for* S *;*
*(4)*  M:F→ℜ *is the marking, and* $M_{0}$ *is the initial marking.*

Compared with Cyber net System, the Generalized Cyber P/N System has three different points:

(1) The weight is changed to the function of S;
(2) The capacity of place is limited;
(3) The marking and weight can be real numbers.

To describe some special relationship, two classes of arcs have been introduced: reading arc and writing arc, as defined below.

**Figure 2:**
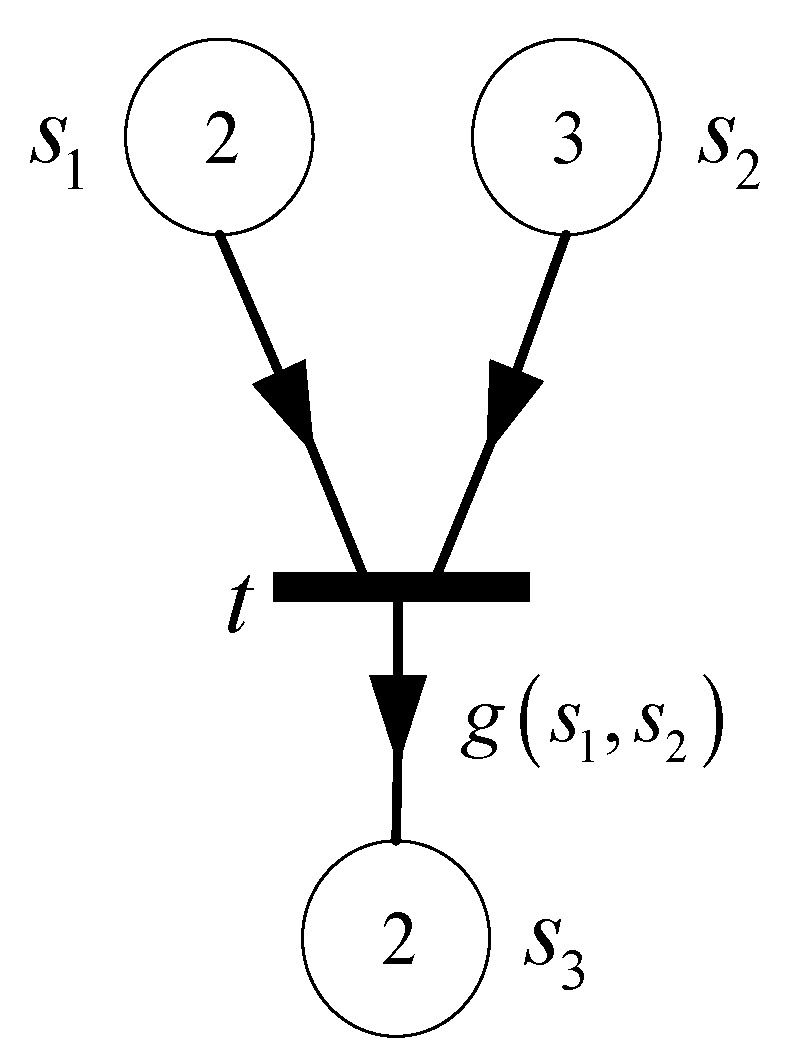
Reading arc and writing arc.

**Definition 8.**  *A directed arc with an arrow in the middle from place (transition) to transition (place) is said to be **reading arc** (**writing arc**) in the Generalized Cyber P/N System. As shown in Figure 2, if arc*(s,t)*is a reading arc, incidence matrix*A(s,t)=0*; and if arc*(t,s)*is a writing arc,*A(s,t)=W(t,s)−s.

**Figure 3:**
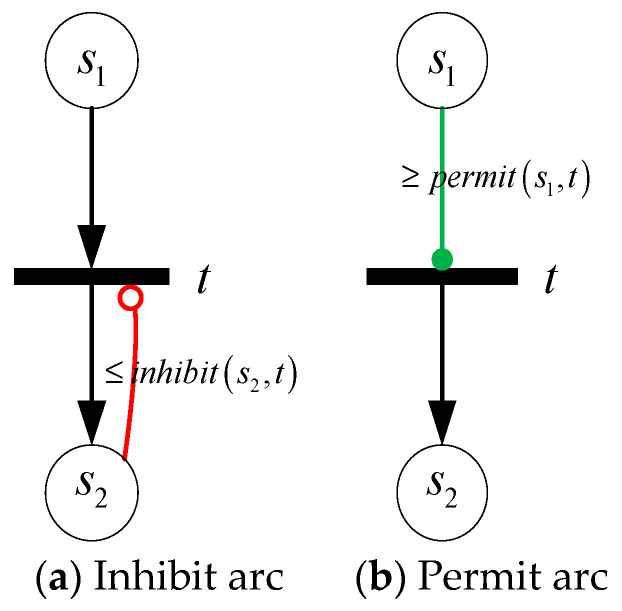
Inhibit arc and permit arc.

In addition, a directed arc from place to transition that uses a hollow (solid) circle instead of an arrow is called an inhibit arc (permit arc). If the marking of place satisfies the function of arc weight, the transition is inhibited (permitted). As shown in Figure 3a, if $M\left(s_{2}\right)\leq inhibit\left(s_{2},t\right)$, transition t can not be fired; and in Figure 3b, if $M\left(s_{1}\right)\geq permit\left(s_{1},t\right)$, transition t can be fired. Different from ordinary transition firing, the marking of input place is invariable for both inhibit arc and permit arc. Note that if multiple inhibit arcs point to the same transition, anyone can stop it; and if multiple permit arcs point to the same transition, it cannot fire until all input places satisfy the weight function.

**Figure 4:**
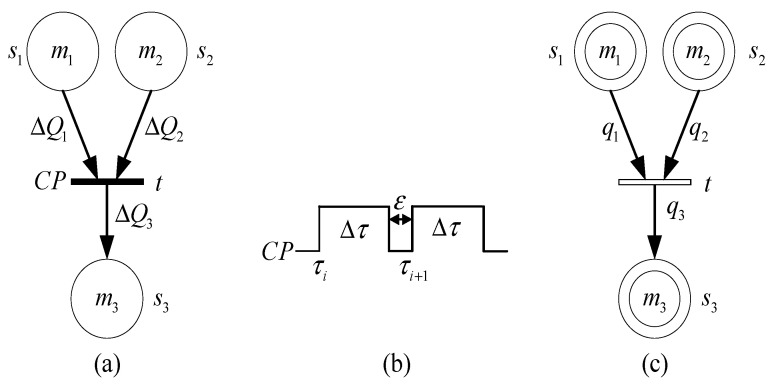
Continuous transition. (**a**) Discrete transition (**b**) timing sequence (**c**) Continuous transition

Consider the model in Figure 4, where t and τ represent the transition and time, respectively. Time event occurs at $\tau_{i}$, that is transition t fires. However, the firing of transition will continue for a while. The firing will stop at $\tau_{i}+\Delta \tau$ when the marking removed from place $s_{1}$ and $s_{2}$ is $\Delta Q_{1}$, $\Delta Q_{2}$, and the incoming marking of place $s_{3}$ is $\Delta Q_{3}$. Accordingly, the mean change of them is $\frac{\Delta Q_{1}}{\Delta \tau }$, $\frac{\Delta Q_{2}}{\Delta \tau }$ and $\frac{\Delta Q_{3}}{\Delta \tau }$, which can be represented as instantaneous velocity of the marking change when Δτ tends to zero, that is $\frac{\Delta Q_{1}}{\Delta \tau }\to q_{1}$, $\frac{\Delta Q_{2}}{\Delta \tau }\to q_{2}$ and $\frac{\Delta Q_{3}}{\Delta \tau }\to q_{3}$. Moreover, $q_{1}$, $q_{2}$ and $q_{3}$ can be a function of a constant or time, also a multivariate function of time and marking. Suppose that ε is a high order infinitesimal amount of Δτ, that is $\tau_{i+1}\to \tau_{i}+\Delta \tau \left(i=0,1,2,\cdots n\right)$. For $\left[\tau_{0},\tau_{n}\right]$, the change amount of $s_{1}$, $s_{2}$ and $s_{3}$ is $\sum_{i=0}^{n}q_{1i}\cdot \Delta \tau$, $\sum_{i=0}^{n}q_{2i}\cdot \Delta \tau$ and $\sum_{i=0}^{n}q_{3i}\cdot \Delta \tau$ respectively. When n→∞, $\sum_{i=0}^{n}q_{1i}\cdot \Delta \tau \to \int_{\tau_{0}}^{\tau_{n}}q_{1}d\tau$, $\sum_{i=0}^{n}q_{2i}\cdot \Delta \tau \to \int_{\tau_{0}}^{\tau_{n}}q_{2}d\tau$, $\sum_{i=0}^{n}q_{3i}\cdot \Delta \tau \to \int_{\tau_{0}}^{\tau_{n}}q_{3}d\tau$. Therefore, transition t can be regarded as continuous firing driven by time events. Such transition is defined as continuous transition, and the symbol is shown in Figure 4c.

**Definition 9.**  *A sextuple* $\sum =\left(S,T;F,K,W,M_{0}\right)$ *is said to be a **Generalized Continuous Cyber Net System** if it satisfied the following conditions:*

*(1)*  N=(S,T;F) *is a directed net, which is named the basic net of* ∑ *;*
*(2)*  $K=\left\{k_{L},k_{H}\right\}$*is the capacity function of*N;
*(3)*  W:F→ℜ∪Exp(S,t) *is the weight function, where* Exp(S,t) *is the set of functional expressions for* S *and* t *;*
*(4)*  M:F→ℜ *is the marking, and* $M_{0}$ *is the initial marking.*

The state equation is: $M\left(t\right)=M\left(t_{0}\right)+\to A\cdot U$, $M\left(t_{0}\right)=M_{0}$, where +→ represents substitute plus, A is incidence matrix, U is the matrix of concurrent sequence $U_{1}U_{2}\cdots U_{k}$. The state equation of the model shown in Figure 4 is:

(6) $$M\left(t\right)=\left[\begin{array}{c} M_{1}\left(t\right)\\ M_{2}\left(t\right)\\ M_{3}\left(t\right) \end{array}\right]=\left[\begin{array}{c} M_{1}\left(t_{0}\right)\\ M_{2}\left(t_{0}\right)\\ M_{3}\left(t_{0}\right) \end{array}\right]+\to \left[\begin{array}{c} -q_{1}\\ -q_{2}\\ q_{3} \end{array}\right]\cdot \left[\int_{t_{0}}^{t}\left(\;\right)d\tau \right]=\left[\begin{array}{c} M_{1}\left(t_{0}\right)-\int_{t_{0}}^{t}q_{1}\left(t\right)d\tau \\ M_{2}\left(t_{0}\right)-\int_{t_{0}}^{t}q_{2}\left(t\right)d\tau \\ M_{3}\left(t_{0}\right)-\int_{t_{0}}^{t}q_{3}\left(t\right)d\tau \end{array}\right]$$

The Hybrid Cyber net System is composed of discrete and continuous parts, among which the discrete part is described by the Generalized Cyber P/N System, and the continuous part is described by the Generalized Continuous Cyber net System. In the graphic representation, discrete transition and continuous transition are represented by solid rectangle and hollow rectangle respectively, and discrete place and continuous place are represented by single circle and double circle respectively. The definition of the Hybrid Cyber Net System will be given below.

**Definition 10.**  *A 10-tuple* $\sum =\left(S,T;F,Rd,Wr,Inhibit,Permit,K,W,M_{0}\right)$ *is said to be a **Hybrid Cyber Petri net System** (HCPNS, if it satisfied the following conditions:*

*(1)*  N=(S,T;F) *is a directed net, which is named the basic net of* ∑ *;*
*(2)*  $S=S_{D}\cup S_{C}$ *,* $S_{D}$ *and* $S_{C}$ *is discrete place and continuous place, respectively;*
*(3)*  $T=T_{D}\cup T_{C}$ *,* $T_{D}$ *and* $T_{C}$ *is discrete transition and continuous transition, respectively;*
*(4)*  F⊆S×T∪T×S *,* Rd⊆S×T *,* Wr⊆T×S *and* F∩Rd∩Wr=Φ *;* F *,* Rd *,* Wr *,* Inhibit *and* Permit *is the flow, read, write, inhibitor and permission relationship;*
*(5)*  $K=\left\{k_{L},k_{H}\right\}$ *is the lower and upper capacity function of* N *, and* $k_{L}:S\to ℜ;k_{H}:S\to ℜ$ *, where* ℜ *is a set of real numbers;*
*(6)*  W:F→ℜ∪Exp(S,τ) *is the weight function, where* Exp(S,τ) *is the set of functional expressions for* τ *;*
*(7)*  M:F→ℜ *is the marking, and* $M_{0}$ *is the initial marking.*

**Definition 11.**  ***The condition that transition t will be fired:***

∀s∈t•:(M(s)−WM(s,t))∈[kL(s),kH(s)]∧∀s∈t•:(M(s)+WM(t,s))∈[kL(s),kH(s)]∧∀s∈tW•:W(t,s)∈[kL(s),kH(s)]

*If transition t is fired, it can be recorded as* M[t> *.*

**Definition** **12.** **Result of transition firing.** *After transition t is fired, the original marking* M *will be changed to* $M^{\prime }$ *, then*

(7) M(s)'={M(s)−W(s,t)M(s)+W(t,s)M(s)−W(s,t)+W(t,s)W(t,s)M(s)s∈t•−t•s∈t•−t•s∈t•∩t•s∈tW•        s∉(t•∩t•∪tW•)

*The dynamic equation of HCNS is:*

(8) $$M\left(t_{n}\right)=M_{0}+A\cdot \left[\int_{t_{0}}^{t_{n}}\left(\;\right)\right]d\tau$$

## 4. HCPNS Modelling Approach of a Master-Slave Charging System

The wireless rechargeable sensor network is a combination of sensor nodes, fixed chargers and a mobile charger. Fixed charging is used as the master mode, and mobile charging as the slave mode. In the Hybrid Cyber Petri net System for MSC, the fixed chargers and a mobile charger are represented by the continuous place $c_{1}\cdots c_{m-1}$ and $c_{m}$. The minimum energy threshold and battery capacity of the mobile charger are represented by $k_{L}\left(c_{m}\right)$, $k_{H}\left(c_{m}\right)$. The sensor nodes with fixed charging are denoted by continuous place $\left\{s_{1}\cdots s_{j}\right\}$ and the nodes with mobile charging is denoted by $\left\{s_{j+1}\cdots s_{n}\right\}$. Similarly, $k_{L}\left(s_{p}\right)$ and $k_{H}\left(s_{p}\right)$ is the minimum energy threshold and battery capacity of node $s_{p}$. Due to space limitation, only part of the chargers and sensor nodes are drawn in the paper.

**Figure 5:**
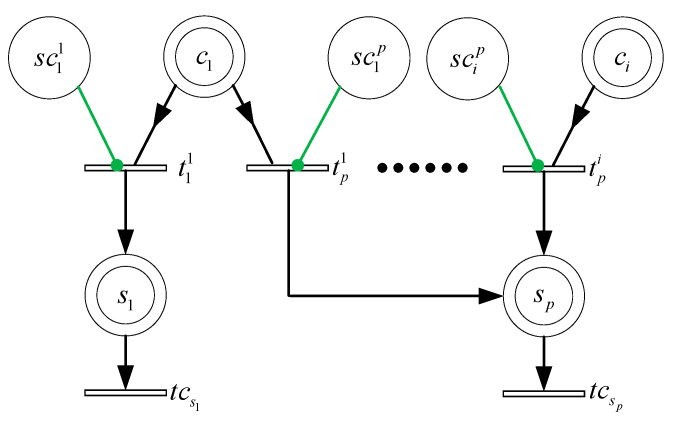
Fixed charging subnet.

**Figure 6:**
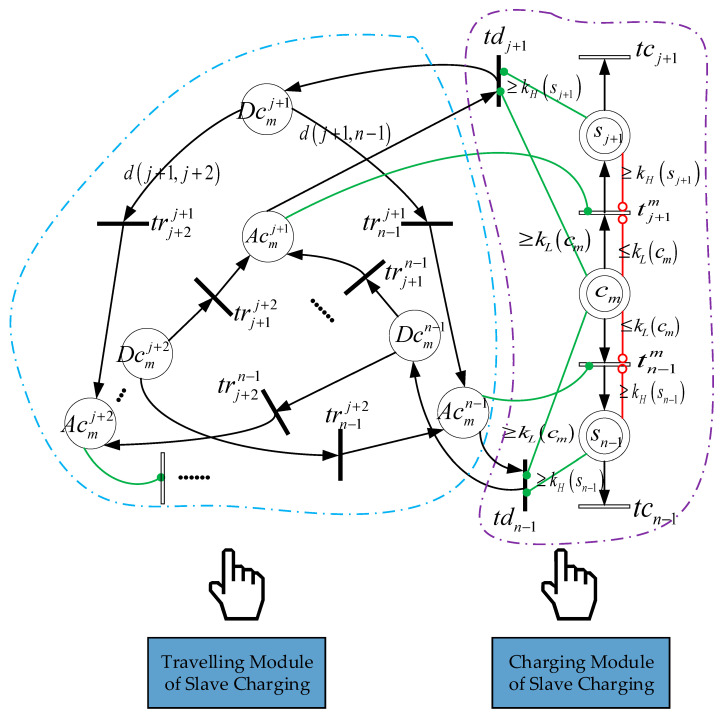
Mobile charging subnet.

Compared to our previous work [8], the model presented in Figure 5 and Figure 6 takes into account new decision parameters of the network. Table 2 and Table 3 allow readers to quickly gain an understanding of the HCPNS model, which contains two subnets representing different functions indicated as follows: (1) The “fixed charging” subnet; and (2) the “mobile charging” subnet. The main function of each subnet is described below.

### 4.1. Fixed Charging Subnet

In the Master-Slave Charging system, most of the energy is obtained by fixed charging. The fixed charging subnet is represented in Figure 5. If the node $s_{p}$ is within the radiation coverage of the fixed charger $c_{i}$, that is marking $M\left(sc_{i}^{p}\right)=1$, the transition $t_{p}^{i}$ is enabled. Since the energy of fixed charger can be replenished at any time, $c_{i}$ and $sc_{i}^{p}$ is connected with $t_{p}^{i}$ by a read arc and a permit arc, respectively. The received power of $s_{p}$ from $c_{i}$ is denoted by $W\left(t_{p}^{i},s_{p}\right)=\frac{\alpha }{{\left(d_{ip}+\beta \right)}^{2}}$, and the consumption power is represented by $W\left(s_{p},tc_{s_{p}}\right)=r_{p}$. From Equation (8),

(9) $$M\left(s_{p}\right)=M_{0}\left(s_{p}\right)+\left\{\left[\sum_{i=1}^{m-1}W\left(t_{p}^{i},s_{p}\right)\cdot M\left(sc_{i}^{p}\right)\right]-W\left(s_{p},tc_{s_{p}}\right)\right\}\cdot T$$

Furthermore, taking into account the AEQ, Equation (9) can be updated as follow.

(10) $$\begin{array}{ccc} M\left(s_{p}\right) & =M_{0}\left(s_{p}\right)+ & min[k_{H}\left(s_{p}\right)-M_{0}\left(s_{p}\right)+W\left(s_{p},tc_{s_{p}}\right)\\ & \cdot & T,\overset{m-1}{\underset{i=1}{\sum }}W\left(t_{p}^{i},s_{p}\right)\cdot M\left(sc_{i}^{p}\right)\cdot T]-W\left(s_{p},tc_{s_{p}}\right)\cdot T\\ & =M_{0}\left(s_{p}\right) & +Q\left(s_{p}\right)-W\left(s_{p},tc_{s_{p}}\right)T \end{array}$$

where $Q\left(s_{p}\right)=min\left[k_{H}\left(s_{p}\right)-M_{0}\left(s_{p}\right)+W\left(s_{p},tc_{s_{p}}\right)\cdot T,\overset{m-1}{\underset{i=1}{\sum }}W\left(t_{p}^{i},s_{p}\right)\cdot M\left(sc_{i}^{p}\right)\cdot T\right]$ is the AEQ of node $s_{p}$.

In the master mode, a large proportion of sensor nodes should be charged, and the more AEQ, the better. Meanwhile, a new charger cannot be deployed until the growth rate of AEQ exceeds a given threshold under cost constraints. Hence, master charging can be formulated as a AEQ maximization problem with constrained growth rate,

(11) $$max\begin{array}{cc} & Q\left(s\right)=\overset{j}{\underset{p=1}{\sum }}Q\left(s_{p}\right) \end{array}$$

(12) $$\mathrm{subject}\;\mathrm{to}\;Q\left(s_{p}\right)\geq W\left(s_{p},tc_{s_{p}}\right)\cdot T,\;\;\delta \geq \delta_{th}$$

where $\delta_{th}$ is a growth rate threshold of AEQ. In addition, it can be seen from the model that simultaneous firing of charging transitions is possible, and the marking of a node’s place can also be added.

### 4.2. Mobile Charging Subnet

This subnet is represented in Figure 6. The control function of the system is performed by using four places denoted by $Ac_{m}^{n}$, $c_{m}$, $Dc_{m}^{n}$, $s_{n}$, four transitions denoted by $t_{n}^{m}$, $tr_{n-1}^{n}$, $td_{n-1}$, $tc_{s_{n}}$ and all of the corresponding arcs. All of these components are interpreted in Table 3. Moreover, the subnet is divided into travelling and charging modules. The former plans the charger’s moving path, and the latter describes the charging behavior. Taking the node $s_{j+1}$ as an example, when the mobile charger reaches this sensor node, that is $M\left(Ac_{m}^{j+1}\right)=permit\left(Ac_{m}^{j+1},t_{j+1}^{m}\right)=1$, the transition $t_{j+1}^{m}$ is fired, which means the node can be charged. When the node is fully charged or the mobile charger is low in power, that is $M\left(s_{j+1}\right)\geq inhibit\left(s_{j+1},t_{j+1}^{m}\right)=k_{H}\left(s_{j+1}\right)$ or $M\left(c_{m}\right)\leq inhibit\left(c_{m},t_{j+1}^{m}\right)=k_{L}\left(c_{m}\right)$, the transition $t_{j+1}^{m}$ is unenabled. In addition, when the node is fully charged and the mobile charger is adequate in power, that is $M\left(s_{j+1}\right)\geq permit\left(s_{j+1},td_{j+1}\right)=k_{H}\left(s_{j+1}\right)$ and $M\left(c_{m}\right)\geq permit\left(c_{m},td_{j+1}\right)=k_{L}\left(c_{m}\right)$, the transition $td_{j+1}$ is firable, $M\left(Ac_{m}^{j+1}\right)=0$ and $M\left(Dc_{m}^{j+1}\right)=1$, which means the mobile charger is able to go to the next node. Theoretically, the charger can leave for all the uncharged node, and the nodes to be visited are determined by the greedy-genetic algorithm to be introduced later. Since the node is charged one-by-one and the distance from the charger is assumed to be 0, the arc weight function $W\left(t_{j+1}^{m},s_{j+1}\right)$ of the received power is set as $0.06\cdot p_{f}$.

After the fixed charging nodes is determined, the set of mobile charging nodes is obtained accordingly, and the energy consumed during the charger’s moving becomes the main variable factor of its energy consumption. Therefore, the slave charging is constructed as a traveling salesman problem in order to minimize the energy consumption of the mobile charger. Assuming constant traveling speed, the mobile charger passes through the shortest path of all nodes only once, taking the base station as the starting point and terminus,

(13) $$min\sum_{\begin{array}{c} u,v=bs,j+1;\\ u\neq v \end{array}}^{n}W\left(Dc_{m}^{u},tr_{v}^{u}\right)$$

(14) $$\mathrm{subject}\;\mathrm{to}\;\sum_{\begin{array}{c} u,v=bs,j+1;\\ u\neq v \end{array}}^{n}Dc_{m}^{u}+Ac_{m}^{v}=1,$$

where the arc function $W\left(Dc_{m}^{u},tr_{n-1}^{v}\right)=d\left(u,v\right)$ represents the distance between two nodes, and Formula (14) ensures that the mobile charger only charges one node at a time.

To sum up, the Master-Slave Charging problem can be formulated:

(15) $$\begin{array}{l} \mathrm{Master}\;\mathrm{Problem}:\;max\;\begin{array}{cc} & Q\left(s\right)=\sum_{p=1}^{j}Q\left(s_{p}\right) \end{array}\\ \mathrm{Slave}\;\mathrm{Problem}:\;min\sum_{\begin{array}{c} u,v=bs,j+1;\\ u\neq v \end{array}}^{n}W\left(Dc_{m}^{u},tr_{v}^{u}\right)\\ \mathrm{subject}\;\mathrm{to}\;Q\left(s_{p}\right)\geq W\left(s_{p},tc_{s_{p}}\right)\cdot T,\;\delta \geq \delta_{th},\\ \mathrm{subject}\;\mathrm{to}\;\sum_{\begin{array}{c} u,v=bs,j+1;\\ u\neq v \end{array}}^{n}Dc_{m}^{u}+Ac_{m}^{v}=1, \end{array}$$

**Figure 7:**
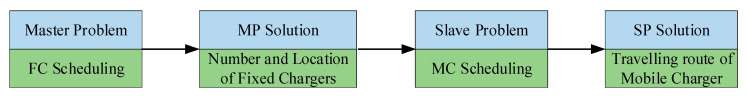
MSC problem-solving process.

The solution process is shown in Figure 7. The number and location of fixed chargers are obtained firstly, and then the travelling route of a mobile charger is determined. For the deployment of fixed chargers, the AEQ of sensor nodes will vary depending on the chargers’ position, when the number of chargers is constant. Therefore, the main problem is to find a set of positions to maximize the AEQ of sensor nodes. It is not hard to see that the construction is a knapsack problem. Furthermore, the slave problem is a typical travelling salesman problem. Both of them are NP-complete, hence traditional optimization methods are difficult to solve it. For this reason, a Greedy-Genetic Algorithm (GGA) is proposed, which has both the global search ability of the genetic algorithm and the local search ability of the greedy algorithm. The greedy nature of the algorithm is mainly reflected in three aspects. First, the number of fixed chargers is gradually increased to find the maximum AEQ within the growth threshold. Second, the traditional crossover operator, where a pair of parents only produce two children, is broken. Instead, multiple crossover operators can be used to produce multiple children, and only the optimal two children evolve into the next generation. Third, individuals are allowed to undergo multiple mutation operations. The standard genetic algorithm process is still used in the greedy-genetic algorithm, and the initial population is randomly generated.

## 5. Simulation and Numeric Results

### 5.1. Simulation Setup and Environment Parameters

A large number of simulations are carried out in this section and numerical results of the algorithm are presented. The simulation is conducted on both small data instances and large data instances. The small data instances include 40 sensor nodes randomly distributed in a two-dimensional area of 400 × 400 m, and the large data instances include 250 sensor nodes randomly distributed in a two-dimensional area of 1000 × 1000 m.

The simulation parameters will be presented in the following. For the fixed charging, we set α=1000 and β=30. The effective radiation radius of a fixed charger is 20 m. The transmitting power of mobile charger is 300 W. For each sensor node, the power consumption rate $r_{p}$ of $s_{p}$ is randomly set among {0.02, 0.03, 0.04 J/s}. The battery capacity of every node is randomly set among {1000, 2000, 3000 J}. The deadline *T* is set to 30 min.

In addition, five criteria are used to describe performance measurements of greedy-genetic algorithm.

(1) *Total AEQ*: The actual electric quantity received by fixed charging. As the objective of master mode, this metric implies the efficiency of our algorithm.
(2) *Proportion of fully-charged sensors*: The ratio of fully-charged sensors to all the sensors.
(3) *Exceeded energy*: Additional energy that the chargers can provide when nodes are fully charged. Since the mobile charger will leave once the node is fully charged, there is no exceeded energy in slave mode.
(4) *Traveling distance of the mobile charger*: The total distance of the mobile charger from and back to the base station.
(5) *Number of dead nodes*: The number of nodes running out of energy. This metric implied the effect of AEQ growth threshold on network performance.

### 5.2. Performance

(1) *Total AEQ:* The total AEQ on both data instances are evaluated by setting the threshold of AEQ growth rate between 2% and 12%. Each different setup is run 10 times, and the average of them will be taken.

**Figure 8:**
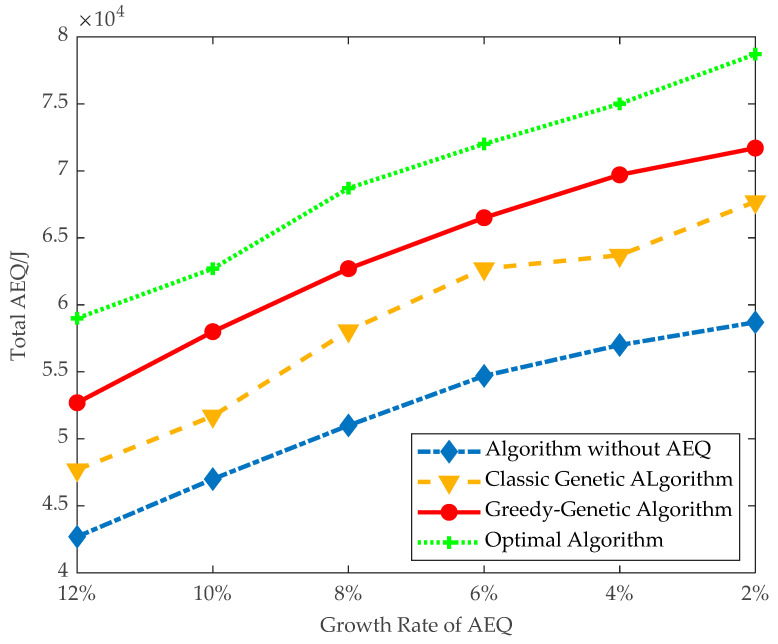
Total AEQ of small data instances.

As shown in Figure 8, GGA achieves greater total AEQ than GGA without AEQ and the classical genetic algorithm, and perform slightly worse than the optimal algorithm. When the threshold of AEQ growth rate is 10%, GGA can achieve 58,000 J total AEQ, up to 92.5% of the AEQ of optimal algorithm. While GGA without AEQ and the classical genetic algorithm only has 53,700 and 47,000 J, being 85.6% and 75% of the AEQ of optimal algorithm, respectively. Similarly, when the threshold of AEQ growth rate is 4%, GGA can achieve 69,700 J total AEQ, up to 93% of the AEQ of optimal algorithm, and another two algorithms only have 64,700 and 57,000 J.

It can also be observed that the trend of total AEQ is increasing along with decreasing the threshold of AEQ growth rate. This is because deploying more fixed chargers is allowed at a greater cost.

**Figure 9:**
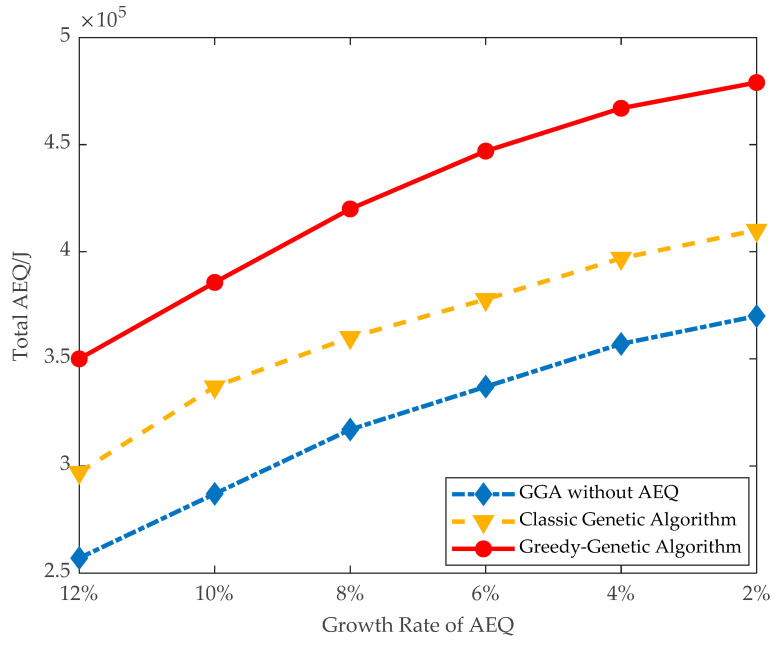
Total AEQ of large data instances.

On the large data instances, all the proposed algorithms, except the optimal algorithm, are conducted, and the observation from Figure 8 still holds here. As shown in Figure 9, the trend of total AEQ is also increasing along with decreasing the threshold of AEQ growth rate.

Overall, Figure 8 and Figure 9 show that GGA can achieve high total AEQ.

(2) *Effectiveness of applying AEQ*: The effectiveness of AEQ is evaluated in terms of the proportion of fully charged and exceeded energy in large instances.

**Figure 10:**
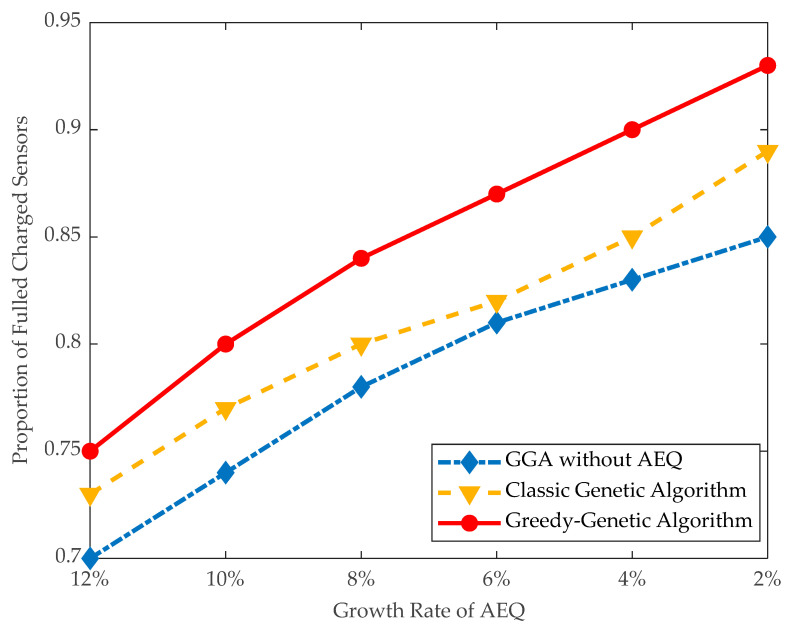
Proportion of fully-charged sensors.

**Figure 11:**
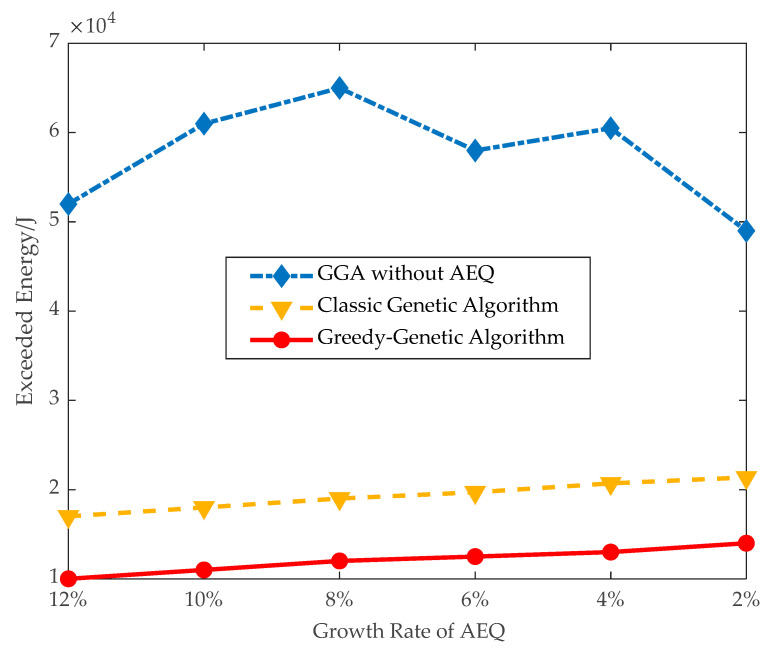
Exceeded energy.

As shown in Figure 10, the proportion of fully-charged sensors using GGA is 84% when the threshold of AEQ growth rate is 8%, and, correspondingly, the exceeded energy shown in Figure 11 is kept at a low level of 12,000 J. Whereas the proportion of fully-charged sensors using GGA without AEQ is 78%, and the exceeded energy is as high as 65,000 J, more than five times of the former. It infers that GGA without AEQ is an energy waste algorithm.

**Figure 12:**
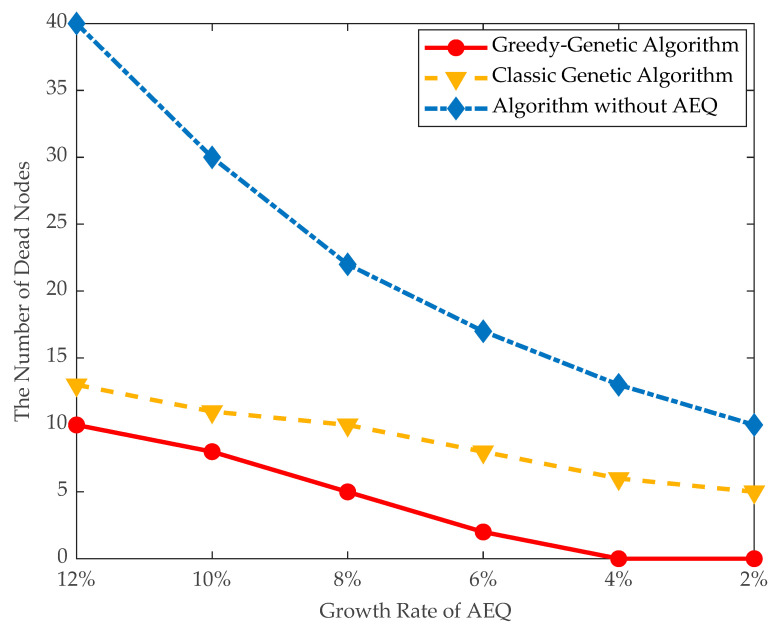
The number of dead nodes.

**Figure 13:**
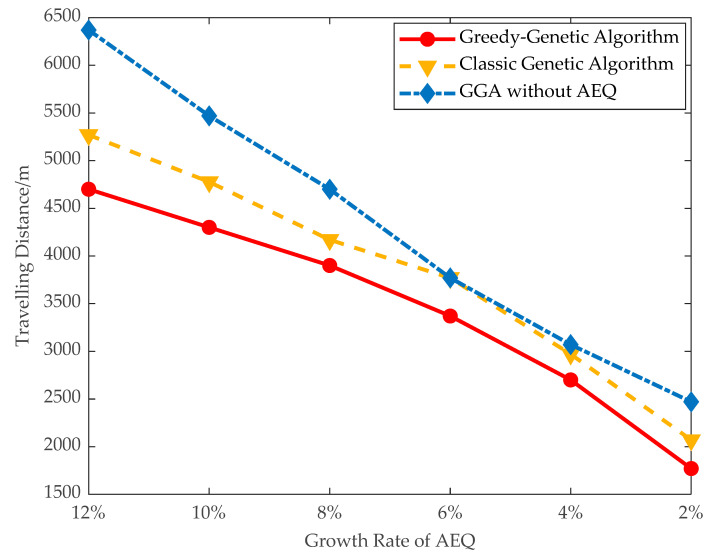
Travelling distance of the charger.

(3) Traveling distance and dead nodes: The traveling distance of the mobile charger and number of dead nodes are evaluated in large data instances. As shown in Figure 12 and Figure 13, when the threshold of AEQ growth rate is 10%, the number of dead nodes is 8 and the traveling distance of the mobile charger is 4300 m. When the threshold of AEQ growth rate is 8%, the traveling distance of the mobile charger is 3900 m and the number of dead nodes is 5. This comparison demonstrates that both the traveling distance and number of dead nodes are turning down with the decrement of the threshold of AEQ growth rate. This is because the lower threshold of the AEQ growth rate allows more fixed chargers to be placed, reducing the number of nodes with mobile charging, thereby easing the burden on the mobile charger and decreasing the waiting time of nodes.

According to the above results, this study presents two reviews from the charging algorithm and scheduling mechanism respectively.

**Review 1:** Compared with the traditional genetic algorithm, the greedy crossover and greedy mutation operator are introduced in this paper, which can improve the defects of weak local search ability and precocity easily. Moreover, the proposed method not only has strong optimization ability, but also the convergence speed is obviously faster than the traditional genetic algorithm, which has a good reference for solving the chargers’ scheduling problem.

**Review 2:** The growth rate of actual electric quantity is introduced to regulate the number of fixed chargers, and the locations of them are determined on the basis of the nodes’ state including the remaining energy, power consumption and position. This scheme not only expands the coverage of fixed chargers, but also reduces the burden of slave charging and ultimately maintains the energy balance of the network.

## 6. Conclusions

This paper proposes the Master-Slave Charging for wireless power supplement in the WRSN firstly. Most of the sensor nodes are replenished by fixed charging, while a few remaining nodes are charged by a mobile charger. Meanwhile, the concept of AEQ is introduced to adjust the scale of fixed chargers; the locations of them are obtained depending on the position, residual energy and power consumption jointly. Secondly, in order to describe charging behavior from mathematical and visual aspects, the classical Petri net is extended and then the Hybrid Cyber Petri net System is proposed. Furthermore, the wireless charging HCPNS model is established to intuitively and accurately describe the energy flow, data flow, position flow and control flow in the MSC. Finally, the greedy-genetic algorithm is applied to simulate and verify the established model from small data instances and large data instances. It can be seen that the performance of GGA is better than the traditional genetic algorithm in terms of total AEQ, the proportion of fully-charged sensors, the traveling distance of the mobile charger and the number of dead nodes. 

There exists a number of interesting open questions related to this paper that deserve future investigation. One important issue is how to develop a more general model that can be used not only for Master-Slave Charging, but also for other charging mechanisms. Secondly, actual electric quantity is not the exclusive index. Moreover, the cost of deployment, impacts on the environment and other factors have to be considered. Thus, how to deploy the chargers under multiple constraints is also a challenging issue.

## Figures and Tables

**Table 1:** Parameters.

Notation	Description
*n*	number of sensor nodes
*m*	number of chargers
$p_{f}$	source power of the charger
$r_{p}$	power consumption rate of sensor node $s_{p}$
$p_{r}\left(s_{p},c_{i}\right)$	received power of $s_{p}$ from $c_{i}$
$E_{c}\left(s_{p}\right)$	consumed energy of $s_{p}$
*E_r_*($s_{p}$)	energy received by $s_{p}$
$E_{need}\left(s_{p}\right)$	energy needed to be full of $s_{p}$
$Q\left(s_{p}\right)$	actual electric quantity of $s_{p}$
δ	growth rate of actual electric quantity
$v_{m}$	energy consumption per time unit of travelling by $c_{m}$
T	charging period of fixed chargers

**Table 2:** Interpretation of place and transition for fixed charging subnet.

Name	Function
$c_{i}$	Represents the charger $c_{i}$.
$sc_{i}^{p}$	Specify whether the sensor node $s_{p}$ is within the radiation of $c_{i}$. It is the case when $M\left(sc_{i}^{p}\right)=1$.
$s_{p}$	Represents the node $s_{p}$. Its marking $M\left(s_{p}\right)$ corresponds to the current energy.
$t_{p}^{i}$	Used to model the charging for $s_{p}$ by the charger $c_{i}$.
$tc_{s_{p}}$	Used to model the consumption of the sensor node $s_{p}$.

**Table 3:** Interpretation of place and transition for mobile charging subnet.

Name	Function
$Ac_{m}^{n}$	Specify whether the mobile charger $c_{m}$ reach the node $s_{n}$. It is the case when $M\left(Ac_{m}^{n}\right)=1$.
$c_{m}$	Represents the mobile charger $c_{m}$. Its marking $M\left(c_{m}\right)$ corresponds to the current energy.
$Dc_{m}^{n}$	Specify whether the mobile charger $c_{m}$ can leave the node $s_{n}$. It is the case when $M\left(Dc_{m}^{n}\right)=1$.
$t_{n}^{m}$	Used to model the charging for $s_{n}$ by the mobile charger $c_{m}$.
$tr_{n-1}^{n}$	Used to model the mobile charger’s travelling from the *n*th to the *n*−1th node.
$td_{n-1}$	Used to model the leaving from the *n*−1th node.

## Data Availability

The data is not applicable due to privacy and ethical restrictions.

## References

[1] Lu X., Wang P., Niyato D., Kim D.I., Han Z. (2016). Wireless charging technologies: Fundamentals, standards, and network applications. IEEE Commun. Surv. Tutor..

[2] Lu X., Wang P., Niyato D., Kim D.I., Han Z. (2015). Wireless networks with RF energy harvesting: A contemporary survey. IEEE Commun. Surv. Tutor..

[3] Bai X., Han W.-Y., Xu L.-J., Zhang J.-W., Li Y.-X. (2020). A radio frequency and vibration energy harvesting antenna based on piezoelectric material. Int. J. Rf Microw. Comput. Aided Eng..

[4] Lai W.Y., Hsiang T.R. (2019). Wireless charging deployment in sensor networks. Sensors.

[5] Madhja A., Nikoletseas S., Raptisab T.P. (2016). Hierarchical, collaborative wireless energy transfer in sensor networks with multiple mobile chargers. Comput. Netw..

[6] Lin C., Lin C., Wu J., Liu Z., Obaidat M.S., Yu W.C., Wu G. (2016). GTCharge: A game theoretical collaborative charging scheme for wireless rechargeable sensor networks. J. Syst. Softw..

[7] Fu L., Cheng P., Gu Y., Chen J., He T. (2016). Optimal charging in wireless rechargeable sensor networks. IEEE Trans. Veh. Technol..

[8] Jiang G.Y., Lam S., Sun Y., Tu L., Wu J. (2017). Joint charging tour planning and depot positioning for wireless sensor networks using mobile chargers. IEEE ACM Trans. Netw..

[9] Wang W., Jing H., Liao J., Yin F., Yuan P., Chen L. (2020). A safe charging algorithm based on multiple mobile chargers. Sensors.

[10] Qin H.Y., Xu L. (2020). Petri-net based modelling and multi-objective optimal deployment for WRSN. Control Eng. Appl. Inform..

[11] Lee J. (2008). A petri net design of command filters for semiautonomous mobile sensor networks. IEEE Trans. Ind. Electr..

[12] Yu Z.H., Fu X., Cai Y., Vuran M.C. (2011). A reliable energy-efficient multi-level routing algorithm for wireless sensor networks using fuzzy petri nets. Sensors.

[13] Ruiz M.C., Mateo J.A., Macia H., Pardo J.J., Olivares T. Formal modelling and performance evaluation of a novel role-based Routing Algorithm for wireless sensor networks. Proceedings of the 2012 18th Annual International Conference on Advanced Computing and Communications (ADCOM), Electronics CityHosur Main Road.

[14] Ruiz M.C., Garrido-Hidalgo C., Gruska D.P., Olivares T., Hortelano D., Roda-Sanchez L. Modeling and evaluation of a power-aware algorithm for IoT bluetooth low energy devices. Proceedings of the 2019 IEEE International Conference on Smart Internet of Things (SmartIoT).

[15] Mostafa A., Hassan K. Robust energy harvesting aware clustering with fuzzy petri net reasoning algorithm. Proceedings of the 2014 IEEE 10th International Conference on Wireless and Mobile Computing, Networking and Communications (WiMob).

[16] Moreno J.C.M., Castro D.M., Ramrez J.L.V. Design of discrete event systems supported on wireless sensors and actuator networks using colored Petri Nets. Proceedings of the 2015 IEEE 2nd Colombian Conference on Automatic Control (CCAC).

[17] Abrishambaf R., Cabral J., Monteiro J., Bal M. An energy aware design flow of distributed industrial wireless sensor and actuator networks. Proceedings of the 2015 IEEE International Conference on Industrial Technology (ICIT).

[18] Jiang F.C., Jou I., Leu F.-Y. (2016). Approaching green sensor field using queue-based optimization technique. J. Netw. Comput. Appl..

[19] Berrachedi A., Boukala-Ioualalen M. Evaluation of the energy consumption and the packet loss in WSNs using deterministic stochastic petri nets. Proceedings of the 2016 30th International Conference on Advanced Information Networking and Applications Workshops (WAINA).

[20] Kostin A.E., Fanaeian Y., Al-Wattar H. (2016). Anycast tree-based routing in mobile wireless sensor networks with multiple sinks. Wirel. Netw..

[21] Le K., Pham B., Tram Q., Bui T., Quan T. CODE-WSN: A formal modelling tool for congestion detection on wireless sensor networks. Proceedings of the 2018 IEEE World Symposium on Communication Engineering (WSCE).

[22] Riouali Y., Benhlima L., Bah S. (2017). Extended batches petri nets based system for road traffic management in WSNs. J. Sens. Actuator Netw..

[23] Mahjoub Y.I., El-Alaouia E.h.C., Nait-Sidi-Mohb A. (2020). Modeling and developing a conflict-aware scheduling in urban transportation networks. Future Gener. Comput. Syst. Int. J. Esci..

[24] He S., Chen J., Jiang F., Yau D.K.Y., Xing G., Sun Y. (2013). Energy provisioning in wireless rechargeable sensor networks. IEEE Trans. Mob. Comp..

[25] Jiang J.R., Liao J.-H. (2016). Efficient wireless charger deployment for wireless rechargeable sensor networks. Energies.

[26] Yao K.H., Jiang J.R., Tsai C.H., Wu Z.S. (2017). Evolutionary beamforming optimization for radio frequency charging in wireless rechargeable sensor networks. Sensors.

[27] Sun G., Liu Y.H., Yang M., Wang A.M., Zhang Y. Charging nodes deployment optimization in wireless rechargeable sensor network. Proceedings of the 2017 IEEE Global Communications Conference.

[28] Yang M., Wang A.M., Sun G., Zhang Y. (2017). Deploying charging nodes in wireless rechargeable sensor networks based on improved firefly algorithm. Comput. Electr. Eng..

[29] Dai H.P., Liu Y., Chen G., Wu X., He T., Liu A.X., Zhao Y. (2018). SCAPE: Safe charging with adjustable power. IEEE ACM Trans. Netw..

[30] Dai H.P., Dai H., Ma H., Liu A.X., Chen G. (2018). Radiation constrained scheduling of wireless charging tasks. IEEE ACM Trans. Netw..

[31] Li L.L., Dai H., Chen G., Zheng J., Dou W., Wu X. (2019). Radiation constrained fair charging for wireless power transfer. ACM Trans. Sens. Netw..

[32] Wan P., Cheng W., Wu B., Wang G. (2019). An algorithm to optimize deployment of charging base stations for WRSN. Eur. J. Wirel. Commun. Netw..

[33] Wang C., Li J., Ye F., Yang Y. (2016). A mobile data gathering framework for wireless rechargeable sensor networks with vehicle movement costs and capacity constraints. IEEE Trans. Comput..

[34] Wang C., Li J., Yang Y.Y., Ye F. (2018). Combining solar energy harvesting with wireless charging for hybrid wireless sensor networks. IEEE Trans. Mob. Comput..

[35] Fu L., He L., Cheng P., Gu Y., Pan J., Chen J. (2016). ESync: Energy synchronized mobile charging in rechargeable wireless sensor networks. IEEE Trans. Veh. Technol..

[36] Shu Y., Shin K.G., Chen J., Sun Y. (2017). Joint energy replenishment and operation scheduling in wireless rechargeable sensor networks. IEEE Trans. Ind. Inform..

[37] Shu Y., Yousefi H., Cheng P., Chen J., Gu Y.J.L., He T., Shin K.G. (2016). Near-optimal velocity control for mobile charging in wireless rechargeable sensor networks. IEEE Trans. Mob. Comput..

[38] Lin C., Wang K., Chu Z.H., Wang K., Deng J., Obaidat M.S., Wu G.W. (2018). Hybrid charging scheduling schemes for three-dimensional underwater wireless rechargeable sensor networks. J. Syst. Softw..

[39] Liu K.Y., Peng J., He L., Pan J.P., Li S., Ling M., Huang Z.W. (2019). An active mobile charging and data collection scheme for clustered sensor networks. IEEE Trans. Veh. Technol..

[40] Sheikhi M., Kashi S.S., Samaee Z. (2019). Energy provisioning in wireless rechargeable sensor networks with limited knowledge. Wirel. Netw..

[41] Ai Z., Liu Y., Song F. (2018). A smart collaborative charging algorithm for mobile power distribution in 5G networks. IEEE Access.

[42] Mo L., Dobre O.A., Ngatched T.M.N., Armada A.G. (2019). Energy-aware multiple mobile chargers coordination for wireless rechargeable sensor networks. IEEE Internet Things J..

[43] Lyu Z.W., Wei Z., Wang X., Fan Y., Xia C., Shi L. (2020). A periodic multinode charging and data collection scheme with optimal traveling path in WRSNs. IEEE Syst. J..

[44] Tian M.Q., Jiao W., Liu J. (2020). The charging strategy of mobile charging vehicles in wireless rechargeable sensor networks with heterogeneous sensors. IEEE Access.

[45] Xie L., Shi Y., Hou Y.T., Lou W., Sherali H.D., Midkiff S.F. (2015). Multi-node wireless energy charging in sensor networks. IEEE ACM Trans. Netw..

[46] Labadi K., Benarbia T., Barbot J., Hamaci S., Omari A. (2015). Stochastic petri net modeling, simulation and analysis of public bicycle sharing systems. IEEE Trans. Autom. Sci. Eng..

